# Photocatalytic Degradation of Methyl Orange and Methylene Blue Dyes by Engineering the Surface Nano-Textures of TiO_2_ Thin Films Deposited at Different Temperatures via MOCVD

**DOI:** 10.3390/molecules28031160

**Published:** 2023-01-24

**Authors:** Zaki S. Khalifa, Mohamed Shaban, Inas A. Ahmed

**Affiliations:** 1 Physics Department, Faculty of Science, Beni-Suef University, Beni-Suef 62511, Egypt; 2 Department of Physics, Faculty of Science, Islamic University of Madinah, Madinah 42351, Saudi Arabia; 3 Nanophotonics and Applications (NPA) Lab, Physics Department, Faculty of Science, Beni-Suef University, Beni-Suef 62514, Egypt; 4 Department of Chemistry, Faculty of Science, King Khalid University, Abha 62224, Saudi Arabia

**Keywords:** anatase TiO_2_, photocatalytic dye removal, surface wettability, reusability, kinetics, specific surface area

## Abstract

TiO_2_ thin films were deposited on quartz substrates by metal–organic chemical vapor deposition (MOCVD) at temperatures of 250, 350, and 450 °C. X-ray diffraction (XRD) data revealed the production of a pure anatase phase, a decrease in crystallite size, and a textural change as deposition temperature increased. Atomic force microscopy (AFM) was used to study the morphological properties and confirm XRD results. UV-Vis.-NIR spectroscopy was used to investigate the optical properties of the samples. The effect of deposition temperature on wettability was investigated using contact angle measurements. Sunlight photocatalytic properties increased with the increase in deposition temperature for methyl orange and methylene blue. Films were post-annealed at 500 °C for 2 h. The effect of annealing on all the above-mentioned properties was explored. The kinetic analysis demonstrated superb agreement with the kinetic pseudo-first-order model. The rate of photocatalytic degradation of MB was ~8, 13, and 12 times that of MO using 250, 350, and 450 °C deposited films, respectively. Photodegradation was found to depend on the specific surface area, type of pollutant, and annealing temperature.

## 1. Introduction

Thin films are preferable for photocatalysis over powders because they can be reused [1]. Titanium dioxide, TiO_2_, is a common candidate for many solar applications because of its availability and compatibility [1,2]. Many factors influence the photocatalytic activity of thin films, for example, crystallite size, crystallinity, orientation, crystalline phase, grain size, roughness, porosity, transparency, and thickness [3,4,5,6,7,8,9,10]. It has been settled that photocatalysis is a synergetic process. However, this did not stop the attempts to study the effect of each parameter independently, which is not an easy task because parameters are cross-linked. For example, Gerbaci et al. [7] tried to study the thickness effect on the photocatalytic activity of TiO_2_ anatase deposited via MOCVD at 400 °C on a soda-lime glass substrate. They observed that beyond 600 nm of thickness, there is a shift in texture and saturation of roughness and crystallite size. Miquelot et al. [8] fixed the thickness and found that specific surface area is the main influencing variable for films deposited via cold-wall MOCVD. Singh et al. [6] found that changing film thickness followed by annealing for RF magnetron sputtered TiO_2_ films resulted in structural and morphological changes with a relatively small optimal film thickness.

Hydrogen production by water splitting, photodecomposition of organic pollutants, and CO_2_ reduction in fuel cells are three main photocatalytic reactions [8,9,10,11]. Adsorption of H_2_O molecules on the surface of the photocatalyst is a prerequisite for these reactions. Adsorption can be molecular or dissociative. Selloni reported that the adsorption type depends on the crystallographic orientation of single crystals [12]. While the high reactive surface (001) shows dissociative adsorption, the lower reactive surface (101) shows molecular adsorption. The (101) facet of anatase has lower surface energy compared to the (001) facet [13,13]. The descending order of TiO_2_ faces according to their surface energy has been reported in many studies [13,13,14,15]. High reactivity is in line with surface energy [12]. However, Pan et al. claimed that the clean facet (101) is more reactive than the (001) facet [16]. Roy et al. [17] reported that the existence of the two facets led to an improved photocatalytic activity as compared to when only one of them was present because facet reduction takes place at (101) and oxidation takes place at (001) due to efficient charge separation. These results are in line with those previously reported by Ohno et al. [18]. Moreover, by modifying the chemistry of preparation, they found that the (112) facet replaced the (001) facet, despite their coexistence [19].

The wettability of a thin film is due to its roughness and/or surface energy. Roughness results from deposition conditions or post-deposition treatment [6,7,8,9,10,20]. According to the Wenzel equation, roughness modifies the degree but not the type of wettability [20]. Surface energy can be triggered because it depends on the chemistry of the surface. For example, UV irradiation increases the number of surface oxygen vacancies, hydroxyl groups, and dangling bonds on the surface of TiO_2_ thin films, which in turn tunes the interfacial energy between the solid surface and the liquid. As a result, the dissociative adsorption of water molecules increases, which raises hydrophilicity [21]. Simultaneously, photons will trigger electron–hole pair formation to reduce or oxidize adsorbents directly or indirectly [10].

Changing substrate temperature to investigate deposition conditions, or annealing, is a common procedure in studying thin films [6,7,8,9,10,22]. In a previous study, we used soda-lime glass substrates and studied the effect of deposition temperature on photodegradation under UV illumination [10]. The photocatalytic activity was found to decrease with the increase in deposition temperature. In this work, we employed quartz substrates to investigate that impact as well as the effects of annealing and pollutant type on photocatalytic activity in daylight.

## 2. Results and Discussion

**Figure 1:**
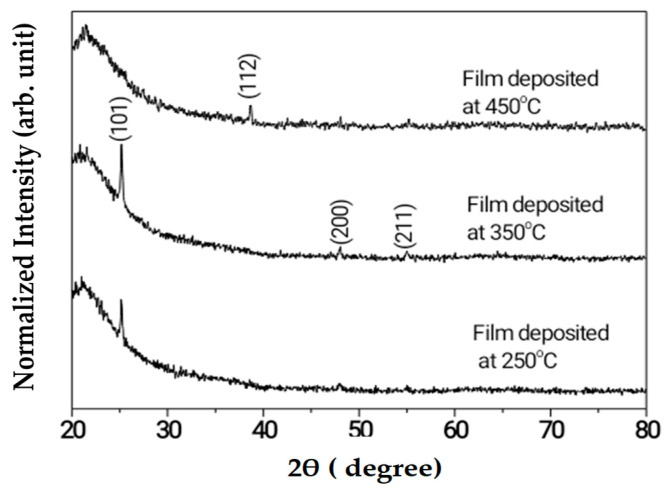
XRD patterns of the as-deposited films.

The structure of the films was explored using XRD. Figure 1 shows the diffractograms at different deposition temperatures. The nature and the limiting parameters of the growth have been studied before [10,23,24]. The obtained crystallite sizes from the (101) peak, which has been considered the most stable face of anatase, were 44 nm and 40 nm for the films deposited at 250 °C and 350 °C, respectively. The texture of the film deposited at 450 °C changed to the less stable face (112). After annealing, films maintained the anatase phase up to 800 °C. Results are not shown here. Texture change with the same Miller indices has been reported for films deposited in a cold MOCVD system as a function of deposition temperature [8]. Other changes in facets have been found for the anatase phase in different MOCVD systems [7,9]. At a constant temperature, texturing developed in the (100) and (211) directions as thickness increases.

**Figure 2:**
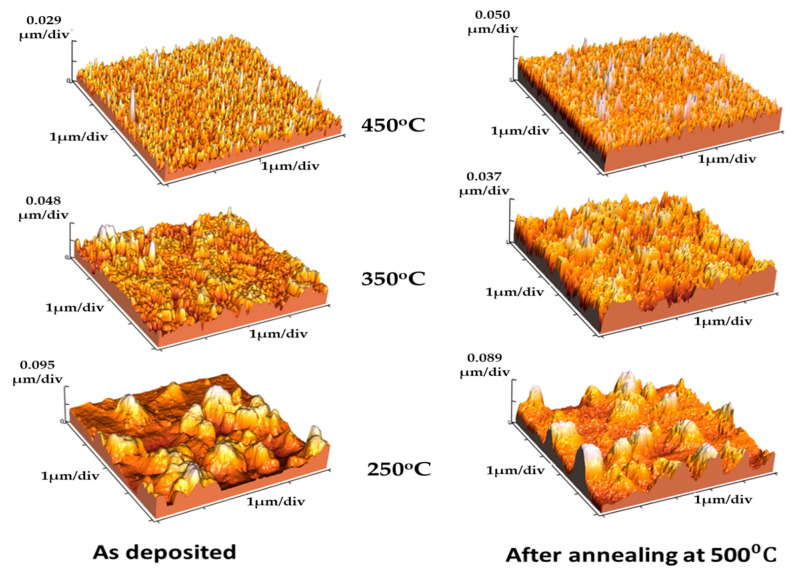
Three-dimensional AFM images of the as-deposited and annealed films.

The morphology of the films was monitored using AFM. Figure 2 represents the 3D pictures before and after annealing at 500 °C. This figure shows that as deposition temperature increased, grain size reduced and porosity increased for as-deposited and annealed films. The average surface roughness was observed to decrease as deposition temperature rose. The related values are shown in Table 1. Similar results employing SEM imaging have previously been published, indicating that films’ structure changes from dense to porous [10]. Porosity has been found to increase with the increase in deposition temperature from optical measurements [23].

**Figure 3:**
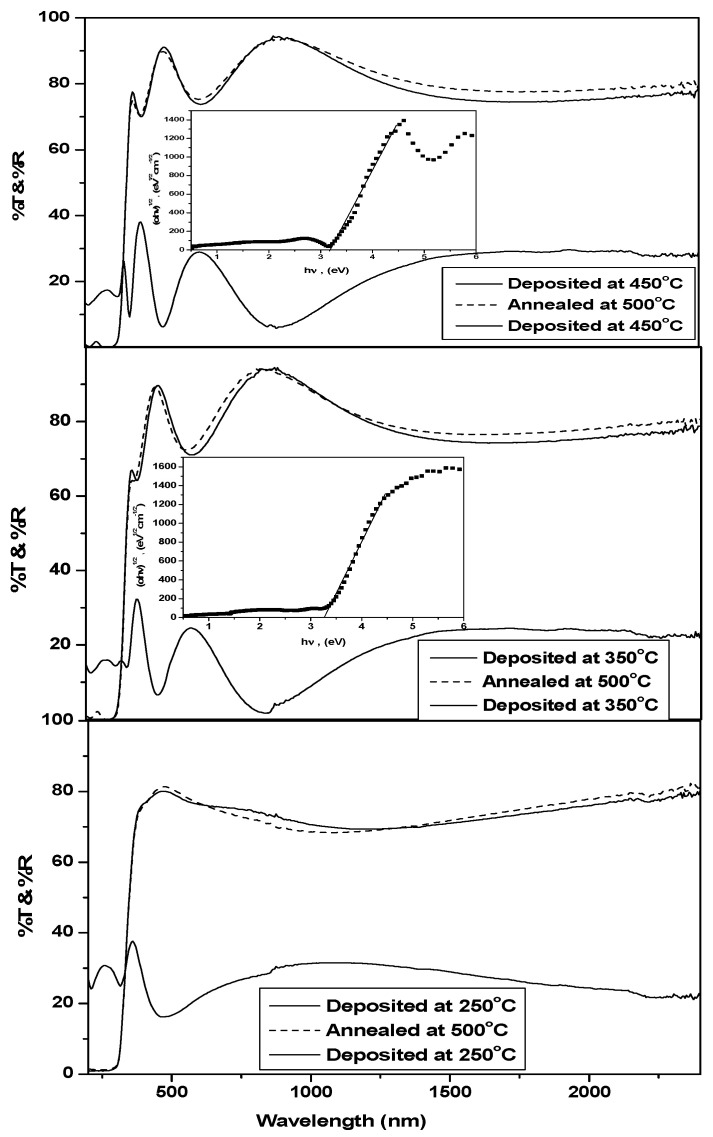
%T of the as-deposited and annealed films and %R of the as-deposited films. Insets show the energy gap calculations of the corresponding films.

Optical properties were investigated through transmittance and reflectance. Annealing does not lead to a noticeable change in the transmitted light. It can be said that all films were transparent. The thickness was found to equal 215 nm and 203 nm for films deposited at 350 and 450 °C, respectively, using the envelope method [23]. The sample deposited at 250 °C was supposed to have a smaller thickness compared to that of the sample deposited at 350 °C, which has been reported for samples deposited under the same conditions [24]. From the following relation,

(1) $$\alpha =\frac{1}{d}lnln\;\frac{\left(1-R\right)}{T}\;$$

the absorption coefficient, *α*, was calculated, where *d* is the thickness, *R* is the reflectance, and *T* is the transmittance of the film [25]. It should be noticed that this relation neglects the interference effect, which was used in the envelope method. The optical band gap can be calculated by using the formula [25]

(2) $${\left(\alpha hע\right)}^{1/2}=\left(hע-E_{g}\right)$$

where *h* is Blanck’s constant and ע is the frequency of incident light [10]. The absorption edge of the film deposited at 250 °C had a close wavelength value to that of the two other films. Therefore, the obtained band gap converged to nearly the same value (3.2 eV), as shown in Figure 3.

**Figure 4:**
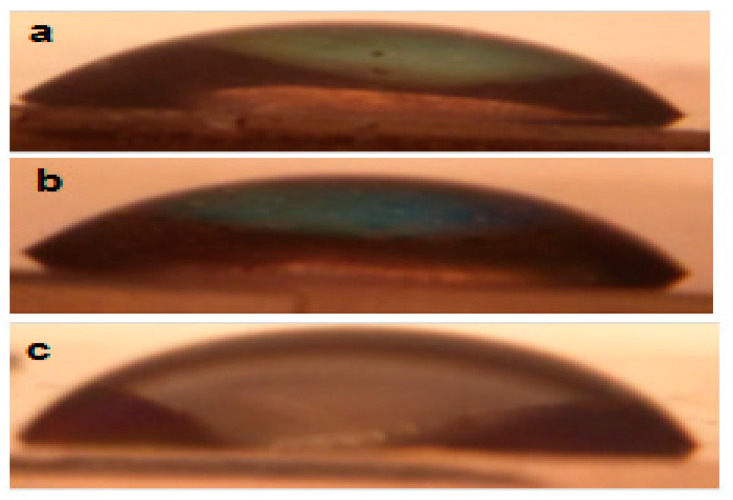
The contact angle of the as-deposited films at (**a**) 250 °C, (**b**) 350 °C, and (**c**) 450 °C.

Contact angle measurements are shown in Figure 4 and the corresponding values are listed in Table 1. It has been reported that contact angle is controlled by surface roughness and surface energy (surface chemistry) [20]. Borras and Gonzalez-Elipe correlated the contact angle and the cross-section morphology, plane morphology, texturing, and surface roughness for microwave-plasma-enhanced CVD TiO_2_ films deposited at 250 °C as a function of temperature for different times [20]. They obtained a direct proportionality between contact angle and surface roughness as a result of increased hydrophobicity. However, they did not attribute it to the Wenzel formula,

(3) $$cos\theta =r\;cos\theta_{o}$$

where *ϴ* is the measured contact angle, *r* is the surface roughness, and *ϴ_o_* is Young’s contact angle [20]. Instead, they reported that the Miwa–Hashimoto formula gave a nearly constant ideal-surface contact angle. They suggested 25 μm^2^ as a minimum area to measure roughness using AFM. Despite this, their findings support the Wenzel formula since roughness improves hydrophobicity.

Generally, roughness increases with low porosity [26]. However, they took different directions in this study. The increase in roughness in the Wenzel equation raised the degree of hydrophilicity. Higher porosity allows water capillary trapping inside pores, which increases hydrophilicity. Therefore, one can say that surface roughness determined the degree of the contact angle in this study [27]. A correlation between contact angle and texture has been suggested before for films deposited via pulsed pressure CVD [9].

**Figure 5:**
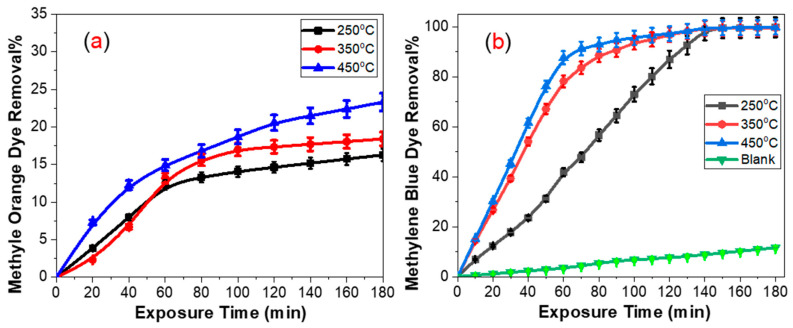
Photocatalytic activity of the as-deposited films toward (**a**) MO and (**b**) MB.

**Figure 6:**
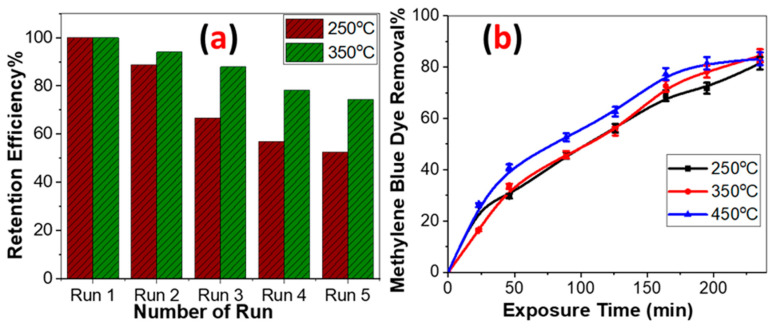
(**a**) Reusability of the samples fabricated at 250 °C and 350 °C before annealing for five runs, and (**b**) reusability of the samples after annealing at 500 °C for 2 h for the catalytic photodegradation of MB.

Photocatalytic activity increases with the increase in deposition temperature for both methyl orange and methylene blue, as shown in Figure 5. Reusability after annealing at 500 °C reduced the photocatalytic activity for the three samples, as shown in Figure 6. In a previous study, we obtained different results [10]. A comparison between the two studies can be conducted as follows. Considering the light source, a UV source was used in the previous study, while sunlight was used in this study. Sunlight contains elements affecting photocatalysis of TiO_2_, such as UV and visible light. UV light reduces the contact angle by tuning surface energy, which enhances photocatalysis and produces e–h pairs that oxidize and reduce undesired molecules directly or indirectly [10,21,27]. In this study, however, the reduction in the contact angle is compensated for at least to some extent by the effect of the visible region, which has been found to recover contact angles with different degrees [20,21,27]. As deposition temperature rises, surface roughness decreases, reducing hydrophilicity. This is anticipated to reduce photocatalytic activity as deposition temperature rises. This is not valid for the two studies because they showed the same trend. Thickness growth is expected to boost photocatalytic activity up to a certain point before saturating or doing the reverse [3,7,9]. This limit varies from one case to the other. It seems that thickness played no role in the two studies because they exhibited the same trend and the saturation limit may not have been reached. For various types of CVD systems, for example, this limit ranges from 395 nm to 900 nm [3,7]. Transparency is important as well. The thickness of a material has a direct impact on its transparency. For thick films, transparency decreases [9]. Thick films with decreased transparency and an insignificant absorption edge exhibit a similar trend to our data [9].

XRD provides us with three contributions that should be discussed: orientation, degree of crystallinity, and crystallite size. Texture change has been assumed to decrease photocatalytic activity when the highest peak intensity changes from (101) to (100) [7]. This has been ascribed to the lower surface energy of the system. This explanation does not agree with the literature, since the (100) surface has higher surface energy, which guarantees enhanced photoreactivity [12,13,13,14,15]. In the study carried out by Miquelot et al. [8], surface energy discussions do not take place despite the texture intensity switching between the (101) and (211) faces. According to Krumdieck et al. [9], planes (101), (200), and (215) diminish as deposition temperature increases. Simultaneously, plane (220) intensifies with the increase in deposition temperature, while other planes, namely (004), (211), and (204), exist at all deposition temperatures. They did not include texturing impact on photocatalytic activity, although they did in explaining hydrophilicity. Stefanov et al. [28,29] quantified the relationship between the ratio of orientation {001}/{101} and the extent of photodegradation for films prepared by reactive DC magnetron sputtering. They estimated a quadratic proportionality between the exposed area of (001) facets and the photodegradation rate as a dependent parameter. Li et al. [30] found enhanced eradication of bacteria and antibiotics by high-activity {001} facets TiO_2_ mounted onto TiO_2_ photoanode. Texture switching from plane (101) for the film deposited at 350 °C to plane (211) of the film deposited at 450 °C is assumed to increase photodegradation because (211) is supposed to be more reactive because of its higher surface energy [15]. Because of the high degree of crystallinity, bulk recombination is reduced, which is expected to increase catalytic activity. Because such surfaces resemble single crystals, a high degree of texturing indicates high crystallinity [27]. Another consideration is the size of the crystallites. As the deposition temperature rises, so does the grain size. This enhances photocatalysis by increasing the surface-to-volume ratio. In other words, when the deposition temperature rises, the specific area increases, which is consistent with the literature [8].

Another parameter that may decrease the photocatalytic activity of films deposited on soda-lime glass substrates is the increase in Na ion diffusion with the increase in deposition temperature [7,31].

The dependence of photocatalytic activity on the type of dye is shown in Figure 5. The photocatalytic activity of the as-deposited films toward MB was higher than that of MO. Photocatalysis takes place in two steps: adsorption on the surface of the metal oxide followed by photodegradation. Adsorption can be due to direct bonding or electrostatic interaction [32]. Direct bonding has been suggested between MO and TiO_2_. This may be due to the sulfonic group, –SO_3_^−^, which can attach to surface Ti(IV) centers through the two sulfonic oxygens [33]. Moreover, it is an electron-withdrawing group that is supposed to decrease photocatalysis [34]. The electrostatic adsorption effect appears upon varying pH values. MB is a cationic dye that is well adsorbed by neutral and basic media. On the other hand, MO is an anionic dye that is well adsorbed by acidic media [33]. Photodegradation involves discoloration and mineralization. In other words, intermediate products play an essential role. For example, Nguyen et al. [35] ascribed the superiority of MB over MO to the increased number of intermediates in the first. In addition, they proved that the intermediates containing the (-N=N-) azo bond are durable and cannot be cleaved easily. Trandafilovi’c et al. [36] compared the photocatalytic degradation between MB and MO on the surface of pure and doped ZnO. Their results agree with this study and that carried out by Nguyen et al. [35]. They used scavengers to study the effect of holes and hydroxyl radicals in photodegradation. They found that both holes and OH radicals play an important role in the case of MB, while in the case of MO holes are the controllers. Five trials were conducted to test the reusability of the films for the removal of 10 ppm MB dye for 100 min each. Before reuse, the samples were washed and dried at 150 °C for 10 min. Figure 6a depicts the retained performance as a function of the number of runs. The results show that after five reusability cycles, the maintained efficiency for 250 °C and 350 °C TiO_2_ films was 52.4% and 74.4%, respectively. The shield generated by dissolved MB molecules on the TiO_2_ surface may reduce the MB removal percentage and therefore the retention efficiency [37]. Figure 6b illustrates the reusability of the samples for the photodegradation of MB after annealing. All samples were annealed at 500 °C for 2 h. The performance of the samples was reduced by almost 25% within 3 h. This demonstrates the acceptable stability of the samples.

**Figure 7:**
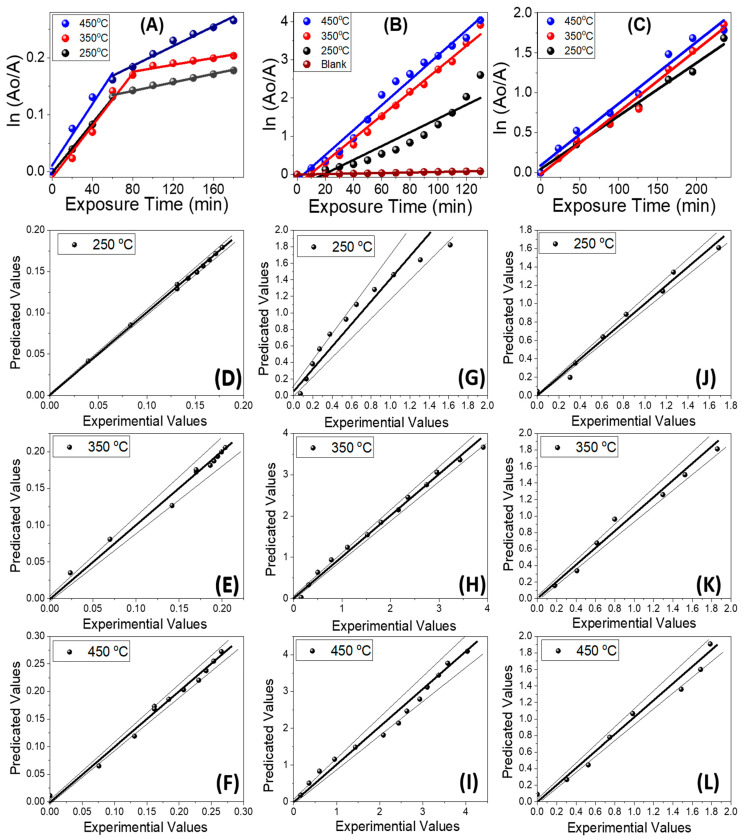
Kinetic study on the catalytic photodegradation of (**A**) MO and (**B**) MB under sunlight using samples before annealing and (**C**) MB using the samples after annealing at 500 °C, and the corresponding parity plots of experimental values and predicted values at (**D**–**J**) 250 °C, (**E**–**K**) 350 °C, and (**F**–**L**) 450 °C.

Several articles indicate that a pseudo-first-order kinetic model is suitable for the reaction kinetic rates of catalytic photodegradation of several dyes [38,39,40,41,42,43]. The rate of reaction, kR, can be obtained from the slope of the linear fitting of the plots, *ln(A*_0_*/A)* vs. exposure time, in Figure 7, based on Equation (4) [43]:

(4) $$\;\;\;\;\;\;\;\;\;\;\;\;\;\;\;\;\;\;\;\;\;\;\;lnln\;\left(A_{0}/A\right)\;=-k_{R}\;t$$

In addition, the statistical parameters, standard deviation, and correlation coefficient (R^2^) were obtained. All the obtained values are reported in Table 2 using the samples before and after annealing. Most of the parameters obtained R_2_ ≥ 0.99, which reflects the superb fitting between the experimental results and this model. Additionally, it can be noticed that there are two values for the reaction rate constant of MO and almost one value for MB in Figure 7A,B and Table 2. The MO photodegradation started with a relatively fast rate change from (220 ± 7) × 10^−5^ to (27 ± 4) × 10^−4^ min^−1^ after the deposition temperature was increased from 250 to 450 °C. This may be ascribed to the direct bonding between MO and TiO_2_, the availability of the active sites, and the anionic nature of MO [32]. In the second stage, t ≥ 60 min, the photodegradation rate was strongly reduced to reach (37 ± 2) × 10^−5^ min^−1^ at 250 °C, and increased to (87± 6) × 10^−5^ min^−1^ at 450 °C. This strong reduction may be ascribed to the reduction in the density of active sites and saturation of the adsorption sites. The value of k_R_ increased from (18 ± 2) × 10^−3^ to (33 ± 1) × 10^−3^ min^−1^ after increasing the deposition temperature from 250 to 450 °C. The rate of photodegradation of MB was ~ 8-, 13-, and 12-fold that of MO at 250, 350, and 450 °C, respectively. This is ascribed to the increase in the number of intermediates, which plays an important role in the photodegradation of MB [41]. Both holes and OH radicals are the main players in the photodegradation of MB, whereas holes are the only main player in the case of MO. The values of k_R_ when using the samples before annealing were almost 3.0-, 4.0-, and 4.5-fold what was obtained using the annealed samples (Figure 7B,C). Note that from Figure 7B, the value of k_R_ = (69 ± 2) × 10^−5^ with R^2^ = 0.9959 for blank MB. Thus, the TiO_2_ films before annealing had the highest catalytic photoactivity and the fastest photodegradation rates for MB dye under sunlight exposure. To represent the errors regarding the fit in a more precise manner, the corresponding parity plots of the experimental values and predicted values are shown in Figure 7D–J for 250 °C, Figure 7E–K for 350 °C, and Figure 7F–L for 450 °C.

Table 3 shows a comparison between the photocatalytic performance of our optimized films and the previously reported TiO_2_-based films in the literature [44,45,46,47,48,49,50,51,52,53,54]. The morphologies and synthesis technique along with the used light source for dye removal are also shown in this table. Our optimized film prepared at 450 °C showed the highest photocatalytic degradation efficiency (97.5%) within only 2 h, although we used sunlight instead of UV light for most of the reported catalysts in Table 3.

## 3. Materials and Methods

A hot-wall MOCVD system was used to prepare the TiO_2_ thin films at 250, 350, and 450 °C. Its specifications have been reported before [24]. XRD measurements of the films were carried out by X-ray diffraction (XRD). θ–2θ scans were recorded using Cu Kα radiation in a Rigaku D-Max B diffractometer (Rigaku D/Max–B, Rigaku, Tokyo, Japan) equipped with a graphite crystal monochromator. AFM characterizations were carried out using an atomic force microscope in the non-contact mode (Park, XE-100E, Park systems, Suwon, South Korea). UV-Vis-NIR spectra were recorded using a 3700 Shimazu double-beam spectrophotometer (Shimazu, Kyoto, Japan). The transmittance spectra were recorded versus air. The static water contact angle measurements were carried out through the sessile drop method in ambient conditions. Approximately 5 mL drop of distilled water was positioned on the surface with a micro-syringe. A CCD camera lens optical system (Canon, Tokyo, Japan) was used to capture digital images of the droplet profile from a location parallel to the films. The photocatalytic activity was measured in sunlight. A total of 10 ppm of methyl orange and methylene blue solutions was used. Absorbance was recorded using a single-beam spectrophotometer (Jenway 7315 UV/Visible, Fisher Scientific, Leicestershire, UK) at 464 nm and 644 nm, respectively. Films were annealed in the air for 2 h.

## 4. Conclusions

Effects of deposition and annealing temperatures were studied for TiO_2_ thin films prepared via MOCVD at 250, 350, and 450 °C on quartz substrates. The pure anatase phase was identified. The crystalline and grain sizes were found to decrease with the increase in the deposition temperature. Photocatalysis is a synergic process. In this study, crystallite and grain sizes played a major role in the process. Texture change may contribute to the enhancement of photocatalysis. The photodegradation of MB was higher and faster than that of MO. Annealing at 500 °C for 2 h reduced photodegradation. The kinetic study showed superb fitting between the experimental results and the pseudo-first-order kinetic model. The rate of photodegradation of MB was ~8-, 13-, and 12-fold that of MO using the deposited films at 250, 350, and 450 °C, respectively.

## Figures and Tables

**Table 1:** Roughness and contact angle for the deposited and annealed samples.

	Projected Area (μm^2^)	Average Roughness (Ra) (nm)		
		250 °C	350 °C	450 °C
As deposited	16	27.15	8.94	3.92
Annealed at 500 °C	25	26.11	9.54	6.59
Contact angle measurements For as-deposited films		39° ± 5°	41° ± 5°	51° ± 5°

**Table 2:** Kinetic rate constants and statistical parameters of photodegradation of MB and MO using MOCVD TiO^2^ films before and after annealing at 500 °C for 2 h.

Sample	MO Before Annealing		MB			
			Before Annealing		After Annealing	
Parameters	k_R_ (min^−1^)	R^2^	k_R_ (min^−1^)	R^2^	k_R_ (min^−1^)	R^2^
250 °C	(220 ± 7) × 10^−5^	0.9990	(18 ± 2) × 10^−3^	0.9437	(67 ± 3) × 10^−4^	0.9933
	(37 ± 2) × 10^−5^	0.9934				
350 °C	(23 ± 2) × 10^−4^	0.9900	(303 ± 9) × 10^−4^	0.9943	(78 ± 4) × 10^−4^	0.9940
	(30 ± 5) × 10^−5^	0.9495				
450 °C	(27 ± 4) × 10^−4^	0.9831	(33 ± 1) × 10^−3^	0.9919	(77 ± 5) × 10^−4^	0.9901
	(87± 6) × 10^−5^	0.9901				

**Table 3:** Comparison of the photocatalytic performance of our optimized photoelectrode with TiO_2_-based catalytic electrodes of different nanomorphologies applied for dye removal in the literature.

Catalyst	Morphology	Synthesize Technique	Dye/Light Source	Removal%	Ref
TiO_2_ sheets	TiO_2_ nanotubes	Electrochemical anodization	180 min UVA irradiation and 4 μM initial dye concentration	74.14% (indigo carmine) 65.71% reactive black 5 (RB5)	[44]
ZnO/TiO_2_ TiO_2_/ZnO TiO_2_	Nanostructured thin film of agglomerated nanoparticles (20 nm)	Sol–gel spin-coating technique	methylene blue (MB) and octadecanoic acid; UV light (6 W)	0.012 min^−1^ (94%) 0.008 min^−1^ (87%) 0.007 min^−1^ (82%)	[45]
TiO_2_/CuO (120 nm/90 nm)	Heterojunction nano-thin films	Magnetron sputtering technology	Rhodamine B (RhB) within 120 min 300 W high-pressure mercury lamp	92.94%	[46]
Tetra(4-carboxyphenyl)porphyrin /Cu Polyoxometalate/TiO_2_	Thin films	Doctor blade technique	100 mL of a 10 mg/L MB, two tubular visible-light lamps, 5 h	49%	[47]
CrMo6/TiO_2_.	Thin films	Doctor blade technique	MB dye UV tubular lamp (7 W, 15 µW/cm^2^), 5 h	83%	[48]
Nb-doped TiO_2_	Thin films of nanoparticles	Sol–gel spin-coating	3 h of visible-light irradiation, 10 ppm of MB	76%	[49]
undoped and P-doped TiO_2_	Films	Spin-coating technique	degradation of MB dye in aqueous solution under UV light (365 nm), 7.1 h	84%	[50]
Pure and TiO_2_, 10%Cu^2+^-doped TiO_2_	Granular structure thin films	Sol–gel dip-coating technique	MB, 180 min, UV-light exposure	92% (0.015 min^−1^) 16% (0.001 min^−1^)	[51]
Ag-loaded TiO_2_-ZnO	Thin films (aggregated nanoparticles of size 20–25 nm)	Dip-coating sol–gel process	methylene blue	80% after 2 h	[52]
cerium oxide-doped rutile TiO_2_	Films	Spray pyrolysis	methyl orange (MO)	0.006 min^−1^	[53]
Ni-doped TiO_2_	Nano-structured thin films (particle size ~92 nm)	Chemical bath deposition method	Ponceau S dye, UV light, and sunlight	~85%	[54]
Pure TiO_2_	Nanotextures of TiO_2_ thin films	MOCVD at 450 °C	Sunlight	97.5% after 2 h	Current work

## Data Availability

Data on the compounds are available from the authors.

## Sample Availability

Samples of the compounds are available from the authors.
